# The Beneficial Effect of Physical Exercise on Cognitive Function in a Non-dementia Aging Chinese Population

**DOI:** 10.3389/fnagi.2019.00238

**Published:** 2019-08-29

**Authors:** Sun Lin, Yang Yang, Qiu Qi, Li Wei, Nie Jing, Zhang Jie, Li Xia, Xiao Shifu

**Affiliations:** ^1^Alzheimer’s Disease and Related Disorders Center, Department of Geriatric Psychiatry, Shanghai Mental Health Center, Shanghai Jiao Tong University School of Medicine, Shanghai, China; ^2^Shanghai I-Zhaohu Senior Care Services Co., Ltd., Shanghai, China; ^3^Key Laboratory of Arrhythmias, Ministry of Education, Shanghai East Hospital, Tong Ji University School of Medicine, Shanghai, China

**Keywords:** exercise, cognitive function, dementia, brain anatomy, lipid

## Abstract

Numerous observational studies have shown that physical exercise promotes cognition in the elderly, however, the results from randomized clinical trials (RCTs) are ambiguous. In addition, potential benefits of exercise in an elderly Chinese population have not been comprehensively addressed. In this study, an investigation was launched which focused on the relationship between physical exercise and cognitive function, blood lipid profiles and brain anatomy in a non-dementia aging Chinese population. A total of 2074 non-dementia elderly subjects were included (self-selected exercise *n* = 1372; self-selected non-exercise *n* = 702). Amongst the subjects, 689 volunteered to receive blood lipid tests, 141 undergo brain magnetic resonance imaging (MRI), and 1399 receive a 1 year cognitive evaluation follow-up. The Beijing version of the Montreal Cognitive Assessment (MoCA) and the Mini-Mental States Examination (MMSE) were used to assess cognitive function. A significant difference in cognitive function was observed at the baseline and during the 1-year follow-up between the self-selected exercise and self-selected non-exercise groups, however, no significant differences in blood lipids and brain anatomy was evident. Physical exercise has a beneficial effect on cognition, particularly visuospatial function, and decreases the risk of dementia in a Chinese aging cohort.

## Background

Aging is an irreversible process and the number of elderly is rapidly increasing. One quarter of the global population will be at least 65 years old in 2020 (Bherer, 2015). Although the occurrence of disability in the elderly is steadily decreasing, the ever-growing population will likely experience a dramatic increase in the prevalence of cognitive dysfunction disorders (Manton et al., 2006). In 2015, approximately 47 million people worldwide were living with dementia. This number is projected to reach 115.4 million by 2050 (Prince et al., 2013), emphasizing the need for a preventative health strategy.

Lifestyle contributes to cognitive function and around 35% of the occurrences of dementia in the elderly are attributable to controllable risk factors including physical inactivity, hypertension, obesity, diabetes, and et al. (Livingston et al., 2017). In this regard, accumulating evidence suggests that exercise has a profound effect on brain plasticity and cognitive function. However, a number of observational studies identified that a negative relationship exists between exercise and the risk of dementia (Livingston et al., 2017). A single meta-analysis of 15 prospective cohort studies involving 33816 non-dementia individuals for 1–12 years follow-up reported that physical activity significantly benefits and maintains cognitive health (Sofi et al., 2011). Furthermore, previous publications had found that exercise and fitness had positive effects on the volume of the hippocampus and CHOL levels (Erickson et al., 2011). Despite these findings, a number of RCTs reported that exercise does not benefit mild cognitive impairment when compared to no interventions or cognitive training in elderly subjects (Legault et al., 2011; Barnes et al., 2013; Sink et al., 2015). Studies conducted in several countries reported the relationship between physical exercise and cognitive or cerebral anatomical changes, but few multi-center studies on non-dementia elderly subjects have been performed on the Chinese population. A 5 years follow-up involving 454 elderly Chinese adults without dementia suggested that prolonged exercise produced positive effects on cognition (Ma et al., 2017). However, more researches focused on cognitive impairment or dementia elderly (Lam et al., 2011; Lam et al., 2012; Ho et al., 2015). Here, in this study, we explored the relationship of physical activity with cognitive function, lipid profiles and brain anatomy in a cohort of non-dementia elderly Chinese individuals. We hypothesized that self-selected exercise (self-reported exercise history) associated with higher cognitive function, lower lipid levels, and higher volumes.

## Subjects and Methods

### Subjects

This was a cross-sectional investigation supported by the National Pillar Program of the China Ministry of Science and Technology (project number: 2009BAI77B03). The study was performed across a range of cities including Shanghai, Beijing, Hefei, Nanchang, Ningbo, Xi’an, and Hangzhou from 2011 to 2012 (Xiao et al., 2013). All subjects were required to meet the following criteria for inclusion in the study: (1) Han Chinese, ≥55 years old; (2) absence of dementia; (3) in accordance with the MiniMental State Examination (MMSE) (Folstein et al., 1975) cutoff score, uneducated subjects ≥18, elementary school educated subjects ≥21, and higher than middle-school educated subjects ≥25; (4) no major medical abnormalities, including nervous system disease or unstable, acute or life-threatening medical ailments; and (5) able to complete the study. A total of 2074 elderly subjects without dementia were included in this study (self-selected exercise *n* = 1372; self-selected non-exercise *n* = 702). Elderly underwent a screening process that included medical history, physical and neurological examinations, and cognitive assessments. All subjects were assessed by clinical physicians to diagnose whether dementia or not through face-to-face interviews. All assessors accepted the consistency training about cognitive function assessments. Life styles including drinking, smoking, tea, and physical diseases including sleep disorder, hypertension, diabetes were all recorded. Out of the subjects, 1399 individuals were willing to be re-assessed after 1 year (self-selected exercise *n* = 915; self-selected non-exercise *n* = 484), 689 individuals accepted baseline blood tests (self-selected exercise *n* = 411; self-selected non-exercise *n* = 278), 141 individuals accepted brain MR imaging (self-selected exercisers *n* = 82; self-selected non-exercisers *n* = 59). Individuals with a history of mental disease or other disorders that could affect cognitive function were excluded. The Beijing version of the MoCA (Nasreddine et al., 2005) and MMSE (Folstein et al., 1975) were used to measure cognitive function. These screening tests consisted of 30 items that measured multiple cognitive domains (including visual space, memory, naming, attention, calculation, abstract, orientation, and language function). The MoCA test contained more attention-executive items than the MMSE. MoCA was sensitive to detect mid cognitive impairment, and MMSE was suited to distinguish dementia.

The subjects were divided into two groups based on exercise history. The definition of exercise in this study referred to ACSM (Garber et al., 2011). We involved self-selected exercisers that met the following criteria. (1) Time: ≥20 min/day; (2) Intensity: moderate intensity (i.e., brisk walking, jogging, climbing stairs, *etc*.) and/or vigorous intensity (i.e., long-distance running, rope skipping, basketball, *etc*.); (3) Frequency ≥4 days/week. The self-selected exercise group was subdivided into two groups based on the cumulative period of: (1) ≥ 10 years and (2) 1–9 years. We involved self-selected non-exercisers that didn’t met the above criteria, including (1) Time: <20 min/day or Intensity: lower than moderate intensity; (2) Frequency <1 day/week. Individuals with uncertain exercise conditions were excluded. Prior to the study, all subjects signed consent forms. Ethical approval was obtained from the Ethics Committee of the Shanghai Mental Health Center.

### Measurement of Blood Indexes

Peripheral blood samples were collected from 7 to 9 a.m. Following an overnight fasting period (≥12 h fasting duration). Clot activating gel-containing serum separator tubes and anticoagulant tubes were used to assay blood indexes. Lipid profile analysis including CHOL, LDL, HDL and triglyceride were measured in Shanghai Mental Health Center.

### MR Image Acquisition and Processing

MR images were acquired using a Siemens Magnetom Verio 3.0T scanner (Siemens, Munich, Germany). T_1_-weighted images were obtained from 176 sagittal slices using 3D magnetization prepared rapid gradient echo acquisition sequence with the following parameters: TR = 2300 ms, TE = 2.98 ms, Flip angle = 9°, spatial resolution = 1^∗^1^∗^1.2 mm^3^.

T_1_-weighted images were processed into surface-based structural data using the automated reconstruction function in the downloaded FreeSurfer version 6.0 software^1^ described by Dale et al. (1999). FreeSurfer was applied to segment brain gray matter, white matter and cerebrospinal fluid, and reconstruct the brain white-gray matter boundary surface. Measurements of cortical thickness, cortical volume, and hippocampus volume for each individual was extracted directly using FreeSurfer.

### Data Analysis

Demographics, lifestyle and physical disease were analyzed using a general linear model test for continuous variables and a χ^2^ test for categorical variables between the different groups. The distinguishing factors between two groups signed with ^∗^ in Table were regressed including demographics, lifestyle and physical disease. Cognitive scores, blood indexes, and brain anatomy indexes were analyzed using general linear models and compared across groups after adjusting for distinguishing factors. Stepwise linear regression analysis was employed using follow-up cognitive function as dependent variable, with self-selected exercise (exercise = 1; non-exercise = 2) as independent variable. Logistic regression analysis was employed using dementia rate of 1 year follow-up as dependent variable, with self-selected exercise (exercise = 1; non-exercise = 2) as the independent variable. Covariates in these models included demographics (age, education, and sex), lifestyle (drinking, smoking, tea) and physical diseases (hypertension and diabetes). SPSS Version 17.0 software with a two-tailed *p-*values of 0.05 was used for all of the statistical analysis.

## Results

**FIGURE 1:**
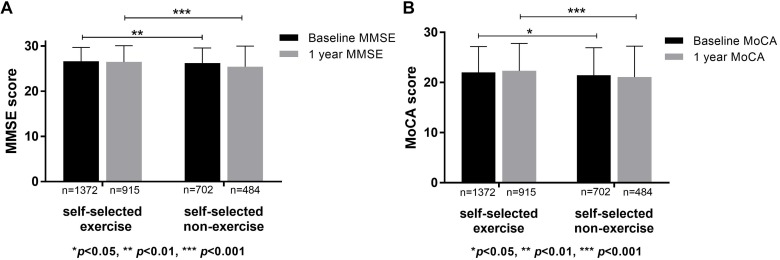
Cognitive functions in self-selected exercise and self-selected non-exercise groups. **(A)** Significant differences were apparent of baseline and 1 year follow-up MMSE scores between self-selected exercise and self-selected non-exercise groups. **(B)** Significant differences of baseline and 1 year follow-up MoCA scores were apparent between self-selected exercise and self-selected non-exercise groups.

Cognitive function between self-selected exercise and self-selected non-exercise groups was compared. Demographics, physical disease, lifestyle, and cognitive scores for self-selected exercisers (*n* = 1372) and self-selected non-exercise (*n* = 702) groups are listed in Table 1. Differences were observed for demographics, lifestyle and physical disease, and the effects of confounding factors (signed by ^∗^ in Table 1) were regressed. Through statistical analysis, higher baseline MMSE and MoCA scores were evident in self-selected exercise group compared to self-selected non-exercise group (*p* < 0.05), which was repeated at 1 year follow-up (*p* < 0.05) (Figure 1). Furthermore, we observed significant differences in visuospatial ability through baseline MoCA, baseline MMSE and 1 year follow-up MMSE tests (*p* < 0.05), whilst no significant difference in memory ability through baseline MoCA, baseline MMSE, 1 year follow-up MoCA tests (*p* > 0.05) except 1 year follow-up MMSE test (*p* < 0.05) between self-selected exercise and self-selected non-exercise groups (*p* > 0.05) was observed. Between ≥10 exercise years and 1–9 exercise years, we observed significant differences in baseline and 1 year follow-up MMSE and MoCA (*p* < 0.05). Furthermore, across all three groups (including ≥10 exercise years, 1–9 exercise years, and non-exercise groups), we observed significant differences in the baseline and 1 year follow-up of MMSE and MoCA (*p* < 0.05).

**TABLE 1:** Demography, life style, physical diseases, and cognitive function in the overall database of study participants in non-dementia elderly Chinese population.

**Characteristic (Baseline)**	**Self-selected exercise^①^**		**Self-selected ^②^**	**① vs.②**	**③ vs. ④**	**② vs. ③ vs. ④**
	**(*n* = 1372)**		**non-exercise**	**F/χ2** **(*P-*value)**	**F/χ2 (*P-*value)**	**F/χ2 (*P-*value)**
			**(*n* = 702)**	**($\eta_{\text{p}}^{2}$)**	**($\eta_{\text{p}}^{2}$)**	**($\eta_{\text{p}}^{2}$)**
	
	**≥10 years^③^**	**1–9 years^④^**				
	**(*n* = 988)**	**(*n* = 384)**				

Age(year)	70.86 ± 7.251	67.77 ± 6.560	70.96 ± 8.163	0.083(0.774)	101.876(0.000^∗^)	45.792(0.000^∗^)
Male/Female	478/510	163/221	304/398	2.184(0.139)	3.910(0.048^∗^)	6.108(0.047^∗^)
Education (year)	7.95 ± 4.648	7.4 ± 4.188	7.40 ± 4.873	3.383(0.066)	4.031(0.045^∗^)	3.606(0.027^∗^)
Smoking (Y/N)	283/705	126/258	212/490	0.033(0.855)	2.297(0.130)	2.324(0.313)
Drinking (Y/N)	221/767	99/285	130/572	6.311(0.012^∗^)	1.801(0.180)	8.207(0.017^∗^)
Tea (Y/N)	493/495	168/216	309/393	3.229(0.072)	4.187(0.041^∗^)	7.429(0.024^∗^)
Sleep disorder (Y/N)	136/852	66/318	114/588	0.827(0.363)	2.580(0.108)	3.334(0.189)
Hypertension (Y/N)	463/525	193/191	340/362	0.071(0.789)	1.280(0.258)	1.351(0.509)
Diabetes (Y/N)	151/837	70/314	108/594	0.182(0.670)	1.776(0.183)	1.980(0.372)
Baseline MoCA	22.07 ± 5.161	21.81 ± 5.128	21.44 ± 5.485	5.272(0.022^∗^)(0.003)	11.028(0.001^∗^)(0.008)	5.822(0.003^∗^)(0.006)
Visual space	3.17 ± 1.512	3.21 ± 1.459	2.992 ± 1.671	6.113(0.013^∗^)(0.003)	1.680(0.195)(0.001)	2.250(0.106)(0.002)
Memory	2.26 ± 1.723	2.14 ± 1.738	2.17 ± 1.680	0.648(0.421)(0.000)	9.669(0.002^∗^)(0.007)	4.073(0.017^∗^)(0.004)
Baseline MMSE	26.64 ± 3.058	26.51 ± 3.141	26.21 ± 3.343	7.335(0.007^∗^)(0.004)	4.407(0.036^∗^)(0.003)	3.993(0.019^∗^)(0.004)
Visual space (0/1)	331/657	105/279	266/436	7.751(0.005^∗^)	4.837(0.028^∗^)	12.435(0.002^∗^)
Memory	5.11 ± 1.010	5.18 ± 0.964	5.06 ± 1.051	2.537(0.111)(0.001)	0.005(0.945)(0.000)	0.662(0.516)(0.001)
**Characteristic (1 year follow-up)**	**Self-selected exercise^**①**^**		**Self-selected ^**②**^**	**① vs. ②**	**③ vs. ④**	**② vs. ③ vs. ④**
	**(*n* = 915)**		**non-exercise**	**F/χ2 (*P-*value)**	**F/χ2 (*P-*value)**	**F/χ2 (*P-*value**)
			**(*n* = 484)**	**($\eta_{\text{p}}^{2}$)**	**($\eta_{\text{p}}^{2}$)**	**($\eta_{\text{p}}^{2}$)**
	
	**≥10 years^**③**^**	**1–9 years^**④**^**				
	**(*n* = 664)**	**(*n* = 251)**				
Age(year)	72.3 ± 7.322	67.71 ± 6.514	71.33 ± 8.198	0.429(0.513)	76.078(0.000^∗^)	34.373(0.000^∗^)
Male/Female	322/342	114/137	212/272	1.886(0.170)	0.691(0.406)	2.579(0.275)
Education (year)	8.04 ± 4.607	7.42 ± 4.103	7.29 ± 4.755	5.121(0.024^∗^)	3.563(0.059)	4.270(0.014^∗^)
Smoking (Y/N)	183/481	82/169	150/334	0.625(0.429)	2.311(0.128)	2.904(0.234)
Drinking (Y/N)	142/522	63/188	84/400	4.924(0.026^∗^)	1.445(0.229)	6.456(0.040^∗^)
Tea (Y/N)	336/328	114/137	208/276	4.893(0.027^∗^)	1.959(0.162)	6.858(0.032^∗^)
Sleep disorder (Y/N)	85/579	41/210	79/405	1.648(0.199)	1.915(0.166)	3.467(0.177)
Hypertension (Y/N)	311/353	141/110	238/246	0.006(0.936)	6.354(0.012^∗^)	6.361(0.042^∗^)
Diabetes (Y/N)	97/567	46/205	70/414	0.333(0.564)	1.910(0.167)	2.284(0.319)
1 Year MoCA	22.33 ± 5.442	22.30 ± 5.536	21.07 ± 6.172	8.742(0.003^∗^)(0.006)	14.881(0.000^∗^)(0.016)	7.120(0.001^∗^)(0.010)
Visual space	3.17 ± 1.585	3.23 ± 1.529	2.94 ± 1.724	2.037(0.154)(0.001)	5.602(0.018^∗^)(0.006)	1.212(0.298)(0.002)
Memory	2.40 ± 1.720	2.46 ± 1.739	2.26 ± 1.736	0.953(0.329)(0.001)	5.431(0.020^∗^)(0.006)	1.112(0.329)(0.002)
1 Year MMSE	26.46 ± 3.624	26.5 ± 3.510	25.40 ± 4.567	16.039(0.000^∗^)(0.011)	9.404(0.002^∗^)(0.010)	9.640(0.000^∗^)(0.014)
Visual space (0/1)	223/441	79/172	209/275	14.140(0.000^∗^)	0.367(0.545)	14.489(0.001^∗^)
Memory	5.15 ± 0.997	5.20 ± 0.924	4.95 ± 1.172	8.037(0.005^∗^)(0.006)	2.600(0.107)(0.003)	4.675(0.009^∗^)(0.007)
1 Year Dementia (Y/N)	32/632	8/243	51/433	19.787(0.000^∗^)	1.161(0.281)	20.585(0.000^∗^)

The incidence of dementia was 40 (4.4%) of the 915 elderly subjects in self-selected exercise group and 51 (10.5%) of 484 elderly subjects in self-selected non-exercise elderly group after 1 year follow-up (*p* < 0.05). However, no significant differences between groups that exercised at least 10 years or more and the 1–9 exercise years was observed (*p* > 0.05).

Stepwise linear regression was used to identify the risk factors for cognitive functions, showing that age (*B* = −0.082, *p* = 0.000), education (*B* = 0.192, *p* = 0.000), tea (tea = 1, non-tea = 2; *B* = −0.316, *p* = 0.024), baseline MMSE scores (*B* = 0.654, *p* = 0.000) and self-selected exercise (exercise = 1, non-exercise = 2; *B* = −0.582, *p* = 0.000) were associated with 1 year follow-up MMSE scores. Furthermore, age (*B* = −0.112, *p* = 0.000), education (*B* = 0.278, *p* = 0.000), (tea = 1, non-tea = 2; *B* = −0.357, *p* = 0.047), baseline MoCA score (*B* = 0.626, *p* = 0.000) and self-selected exercise (exercise = 1, non-exercise = 2; *B* = −0.530, *p* = 0.005) were associated with 1 year follow-up MoCA scores. Finally, logistic regression analysis revealed that education (*B* = 0.089, Wald = 11.632, *p* = 0.000) was positively associated with dementia occurrence after 1 year, however, age (*B* = −0.079, Wald = 25.964, *p* = 0.000), and self-selected non-exercise (*B* = −0.829, Wald = 13.386, *p* = 0.001) negatively affected the dementia rate at 1 year follow-up.

### Blood Lipid Profiles Between Self-Selected Exercisers and Self-Selected Non-exercise Groups

Blood lipid tests were obtained from a total of 689 individuals in the self-selected exercise (*n* = 411) and self-selected non-exercise (*n* = 278) groups extracted from the whole database (Table 2). The effects of distinguishing factors between groups were regressed (signed by ^∗^ in Table 2). Through regular statistical analysis, no changes in TG, CHOL, HDL and LDL levels was observed between self-selected exercise and self-selected non-exercise groups (*p* > 0.05). No significant differences in blood lipid levels was found in the group that exercised at least 10 years or more and the group that exercised from 1 to 9 year, or across the three groups (*p* > 0.05).

**TABLE 2:** Demography, life style, physical diseases, and lipid profile of study participants in the database subgroup on a non-dementia elderly Chinese population.

**Characteristic**	**Self-selected exercise^①^**		**Self-selected**	**① vs. ②**	**③ vs. ④**	**② vs.③ vs. ④**
	**(*n* = 411)**		**non-exercise^②^**	**F/χ^2^(*P-*value)**	**F/χ^2^(*P-*value)**	**F/χ^2^(*P-*value)**
			**(*n* = 278)**	**($\eta_{\text{p}}^{2}$)**	**($\eta_{\text{p}}^{2}$)**	**($\eta_{\text{p}}^{2}$)**
	
	**≥10 years^③^**	**1–9 years^④^**				
	**(*n* = 306)**	**(*n* = 105)**				
Age(year)	72.42 ± 7.518	68.04 ± 7.071	71.27 ± 8.147	0.002(0.961)	27.337(0.000^∗^)	12.603(0.000^∗^)
Male/Female	137/169	45/60	119/159	0.147(0.701)	0.116(0.733)	0.263(0.877)
Education (year)	8.01 ± 4.440	8.90 ± 3.799	7.54 ± 4.527	4.168(0.042^∗^)	3.433(0.065)	3.731(0.024^∗^)
Smoking (Y/N)	77/229	28/77	76/202	0.275(0.600)	0.093(0.761)	0.366(0.833)
Drinking (Y/N)	60/246	18/87	46/232	0.664(0.415)	0.309(0.578)	0.986(0.611)
Tea (Y/N)	142/164	36/69	101/177	3.351(0.067)	4.677(0.031^∗^)	8.116(0.017^∗^)
Sleep disorder (Y/N)	49/257	20/85	59/219	2.156(0.142)	0.515(0.473)	2.632(0.268)
Hypertension (Y/N)	144/162	55/50	135/143	0.001(0.971)	0.887(0.346)	0.888(0.641)
Diabetes (Y/N)	48/258	26/79	36/242	3.159(0.076)	4.362(0.037^∗^)	7.958(0.019^∗^)
Blood Lipid Profile						
TG (mmol/L)	1.83 ± 1.347	1.92 ± 1.566	1.73 ± 1.237	0.974(0.324)(0.001)	0.039(0.844)(0.000)	0.527(0.591)(0.002)
CHOL (mmol/L)	4.84 ± 1.083	4.80 ± 1.035	4.88 ± 1.088	0.196(0.658)(0.000)	0.160(0.690)(0.000)	0.102(0.903)(0.000)
HDL (mmol/L)	1.21 ± 0.313	1.21 ± 0.361	1.23 ± 0.340	0.023(0.879)(0.000)	0.196(0.659)(0.000)	0.266(0.766)(0.001)
LDL (mmol/L)	2.89 ± 0.848	2.85 ± 0.911	2.97 ± 0.8883	1.356(0.245)(0.002)	0.556(0.456)(0.001)	0.668(0.513)(0.002)

*TG, triglyceride; CHOL, cholesterol; HDL, high density lipoprotein; LDL, low density lipoprotein.*

### Brain Anatomy Between Self-Selected Exercise and Self-Selected Non-exercise Groups

Brain MR images were obtained from 141 individuals from the whole database. The demographics, physical disease, and lifestyle for the self-selected exercise (*n* = 82) and self-selected non-exercise (*n* = 59) groups were listed in Table 3. The effects of distinguishing factors were regressed (signed by ^∗^ in Table 3). No significant differences in regional cortical thickness (a total of 68 brain region, not listed in table), cortical volume, and hippocampus volume, occurred between self-selected exercise and self-selected non-exercise, the group that exercised at least 10 or more years and the group that exercised 1–9 years, or across all three sub-groups (*p* > 0.05).

**TABLE 3:** Demography, life style, physical diseases, and brain anatomy of study participants in the database subgroup on a non-dementia elderly Chinese population.

**Characteristic**	**Self-selected exercise^①^**		**Self-selected**	**① vs. ②**	**③ vs. ④**	**② vs. ③ vs. ④**
	**(*n* = 82)**		**non-exercise^②^**	**F/χ^2^(*P-*value)**	**F/χ^2^(*P-*value)**	**F/χ^2^(*P* value)**
			**(*n* = 59)**	**($\eta_{\text{p}}^{2}$)**	**($\eta_{\text{p}}^{2}$)**	**($\eta_{\text{p}}^{2}$)**
	
	**≥10 years^③^**	**1–9 years^④^**				
	**(*n* = 55)**	**(*n* = 27)**				
Age (year)	69.11 ± 6.839	65.41 ± 5.719	68.25 ± 7.473	0.092(0.762)	5.881(0.018^∗^)	2.636(0.075)
Male/Female	30/25	11/16	29/30	0.010(0.921)	1.380(0.240)	1.390(0.499)
Education (year)	9.84 ± 3.207	10.19 ± 3.352	9.93 ± 3.3398	0.001(0.973)	0.208(0.650)	0.101(0.904)
Smoking (Y/N)	13/42	5/22	21/38	3.191(0.074)	0.277(0.599)	0.454(0.797)
Drinking (Y/N)	11/44	5/22	9/50	0.426(0.514)	0.025(0.874)	0.454(0.797)
Tea (Y/N)	24/31	8/19	23/36	0.000(0.996)	1.493(0.222)	1.493(0.474)
Sleep disorder (Y/N)	2/53	5/22	13/46	5.135(0.023^∗^)	5.137(0.023^∗^)	8.430(0.015^∗^)
Hypertension (Y/N)	27/28	14/13	28/31	0.089(0.766)	0.055(0.814)	0.144(0.931)
Diabetes (Y/N)	7/48	5/22	9/50	0.010(0.919)	0.486(0.486)	0.490(0.783)
Volume (cm^3^)						
Total volume	1459.20 ± 141.52	1422.33 ± 158.53	1475.69 ± 143.472	2.072(0.152)(0.015)	0.556(0.458)(0.007)	1.390(0.253)(0.020)
Cortex volume	417.28 ± 37.389	408.99 ± 38.037	420.35 ± 38.783	0.057(0.811)(0.000)	0.825(0.367)(0.011)	0.034(0.967)(0.000)
Left hippocampus	3.64 ± 0.338	3.66 ± 0.375	3.65 ± 0.472	0.281(0.597)(0.002)	0.001(0.975)(0.000)	0.600(0.550)(0.009)
Right hippocampus	3.88 ± 0.404	3.92 ± 0.398	3.84 ± 0.426	2.175(0.143)(0.016)	0.000(0.984)(0.000)	1.713(0.184)(0.025)

## Discussion

In this study, three major findings were presented. First, non-dementia elderly individuals with self-selected exercise showed significantly higher cognitive performance and a lower dementia rate of 1 year follow-up compared to those with self-selected non-exercise. Next, no significant difference in blood lipids occurred in response to self-selected exercise in elderly individuals. Finally, no significant differences in brain anatomy occurred between self-selected exercise and self-selected non-exercise in elderly individuals.

This study highlighted higher cognitive performance in self-selected exercisers without dementia, supporting previous observational studies (Hamer and Chida, 2009; Prince et al., 2013; Livingston et al., 2017). Stepwise linear regression analysis also suggested that, besides age and education, exercise was an important risk factor for the occurrence of dementia in the elderly. A meta-analysis of prospective studies supported this concept and suggested that physical exercise lowered the risk of cognitive deficits and dementia by up to 38% (Sofi et al., 2011), which supported the findings of this research study. After 1 year follow-up, the rate of dementia occurrence in the self-selected exercise group (4.4%) was significantly lower than the self-selected non-exercise group (10.5%) in the present study. Our cohort was non-dementia aging Chinese population including normal cognitive and mild cognitive impairment elderly. Most subjects converting into dementia with 1 year were mild cognitive impairments elderly. Furthermore, we found better visuospatial ability with self-selected exercisers, which was similar with some previous publications. Aerobic exercise may have a positive effect on improving a potential benefit on visuospatial domain of cognition and et al., in stroke survivors (Zheng et al., 2016). Improvements in visual ability and et al., in the stabilization exercise training suggest exercise for the treatment of idiopathic scoliosis to improve internal body orientation (Yagci et al., 2018). A serious of RCTs reported that physical exercise did not improve cognitive function or lower the risk of dementia (Legault et al., 2011; Barnes et al., 2013; Sink et al., 2015). This finding might be related to the short study period. All of the subjects involved in the present study maintained exercise for at least 1 year and 97% of the subjects maintained exercise for two or more years. The longest intervention duration was less than 2 years in the previous RCT (Lam et al., 2011), which suggested that exercise duration of two or more years was an important influencing factor on cognition. Significant differences in cognition function were observed including a baseline and 1 year follow-up MMSE and MoCA of the group that exercised 10 or more years and the group that exercised 1–9 years, demonstrating that the duration of physical exercise affected cognition (Varma et al., 2015). However, no differences in dementia occurrence between the 10 or more years and 1—9 exercise years groups, suggesting that the cut-off value of 10 years did not influence the incidence of dementia.

Physical exercise had been shown to have a beneficial impact on dyslipidemia and numerous studies had reported that physical exercise combined with weight loss significantly reduced blood CHOL, LDL, and TG, while improving HDL (O’Donovan et al., 2005; Pattyn et al., 2013; Gordon et al., 2014). However, we observed no significant difference in blood lipid profiles between self-selected exercise and self-selected non-exercise groups. Interventional research to directly assess the impact of training intensity on lipid profiles by controlling training volume showed that significant improvements occurring only in high-intensity compared to moderate intensity groups (Mann et al., 2014). The subjects included in self-selected exercise groups maintained moderate and/or vigorous exercise intensity, not high intensity exercise.

No difference in brain anatomy including regional cortical thickness, total cortical volume, hippocampus volume was found between self-selected exercise and self-selected non-exercise groups. Other studies found that exercise training increased gray matter volume in the prefrontal lobe (Colcombe et al., 2006), temporal lobe, and hippocampus (Erickson et al., 2011). In this study, no region was found to be significantly thicker or larger in the self-selected exercise group. This might be due to the variety of exercise types that the test subjects adopted in the different cohorts. The previous studies demonstrated that significant increases in brain volume were found as a function of aerobic fitness training but were not found in stretching and toning (non-aerobic) (Colcombe et al., 2006; Erickson et al., 2010). In the current research, the exercise aerobic and anaerobic exercise types were not distinguished, which could cause the negative brain anatomy results.

There were several study limitations. Follow-up tests using MR imaging and blood indexes were not performed, and therefore, could not directly show causality of exercise on brain anatomy and lipid profiles, either beneficial or harmful. A lack of detailed information regarding oxygen consumption, heart rate and exercise types also limited the description of exercise status. Self-selected exercise based on self-reported by subjects, and several factors including education, lifestyle and comorbid diseases might be association with self-selected exercise, which is a possible bias of the study. The sample size of MR images was significantly smaller than the overall database since only elderly subjects in Shanghai were able to receive MR imaging scans. The follow-up duration was only 1 year and inadequate to reflect dementia occurrence, and we would go on research the cohort in the future.

## Conclusion

The study results demonstrate that physical exercise has beneficial effects on cognition, particularly visuospatial function, and lowers the risk of dementia. No differences in the blood lipids and brain anatomy were observed between self-selected exercise and self-selected non-exercise groups in the Chinese aging cohort.

## Consent to Publication

All subjects also gave written informed consent for the publication of this case report.

## Data Availability

The data supporting our findings can be requested by email to the correspondence author.

## Ethics Statement

This study was carried out in accordance with the recommendations of the “Shanghai Mental Health Center ethical standards committee on human experimentation” with written informed consent from all subjects. All subjects gave written informed consent in accordance with the Declaration of Helsinki. The protocol was approved by the “Shanghai Mental Health Center ethical standards committee.”

## Author Contributions

SL analyzed the data and wrote the manuscript. YY, QQ, LW, NJ, and ZJ evaluated the subjects and collected the data. XS and LX designed the experiment and monitored the quality of the experiment.

## Conflict of Interest Statement

The authors declare that the research was conducted in the absence of any commercial or financial relationships that could be construed as a potential conflict of interest.

## Footnotes

1 http://surfer.nmr.mgh.harvard.edu/

## Abbreviations

ACSM: American College of Sports Medicine
CHOL: cholesterol
HDL: high density lipoprotein
LDL: low density lipoprotein
MMSE: Mini-Mental States Examination
MoCA: Montreal Cognitive Assessment
RCTs: randomized clinical trials
TG: triglyceride.

## References

[B1] Barnes D. E. Santos-Modesitt W. Poelke G. Kramer A. F. Castro C. Middleton L. E. (2013). The Mental Activity and eXercise (MAX) trial: a randomized controlled trial to enhance cognitive function in older adults. *JAMA Int. Med.* 173 797–804. 10.1001/jamainternmed.2013.189 23545598 PMC5921904

[B2] Bherer L. (2015). Cognitive plasticity in older adults: effects of cognitive training and physical exercise. *Ann. N.Y. Acad. Sci.* 1337 1–6. 10.1111/nyas.12682 25773610

[B3] Colcombe S. J. Erickson K. I. Scalf P. E. Kim J. S. Prakash R. McAuley E. (2006). Aerobic exercise training increases brain volume in aging humans. *J. Gerontol. Series A Biol. Sci. Med. Sci.* 61 1166–1170. 10.1093/gerona/61.11.1166 17167157

[B4] Dale A. M. Fischl B. Sereno M. I. (1999). Cortical surface-based analysis. I. Segmentation and surface reconstruction. *NeuroImage* 9 179–194. 9931268 10.1006/nimg.1998.0395

[B5] Erickson K. I. Raji C. A. Lopez O. L. Becker J. T. Rosano C. Newman A. B. (2010). Physical activity predicts gray matter volume in late adulthood: the cardiovascular health study. *Neurology* 75 1415–1422. 10.1212/WNL.0b013e3181f88359 20944075 PMC3039208

[B6] Erickson K. I. Voss M. W. Prakash R. S. Basak C. Szabo A. Chaddock L. (2011). Exercise training increases size of hippocampus and improves memory. *Proc. Natl. Acad. Sci. U.S.A.* 2011 3017–3022. 10.1073/pnas.1015950108 21282661 PMC3041121

[B7] Folstein M. F. Folstein S. E. McHugh P. R. (1975). Mini-mental state. A practical method for grading the cognitive state of patients for the clinician. *J. Psychiatric Res.* 12 189–198. 10.1016/0022-3956(75)90026-6 1202204

[B8] Garber C. E. Blissmer B. Deschenes M. R. Franklin B. A. Lamonte M. J. Lee I. M. (2011). American College of sports medicine position stand. Quantity and quality of exercise for developing and maintaining cardiorespiratory, musculoskeletal, and neuromotor fitness in apparently healthy adults: guidance for prescribing exercise. *Med. Sci. Sports Exercise* 43 1334–1359. 10.1249/MSS.0b013e318213fefb 21694556

[B9] Gordon B. Chen S. Durstine J. L. (2014). The effects of exercise training on the traditional lipid profile and beyond. *Curr. Sports Med. Rep.* 13 253–259. 10.1249/JSR.0000000000000073 25014391

[B10] Hamer M. Chida Y. (2009). Physical activity and risk of neurodegenerative disease: a systematic review of prospective evidence. *Psychol. Med.* 39 3–11. 10.1017/S0033291708003681 18570697

[B11] Ho R. T. Cheung J. K. Chan W. C. Cheung I. K. Lam L. C. (2015). A 3-arm randomized controlled trial on the effects of dance movement intervention and exercises on elderly with early dementia. *BMC Geriatrics* 15:127. 10.1186/s12877-015-0123-z 26481870 PMC4615324

[B12] Lam L. C. Chau R. C. Wong B. M. Fung A. W. Lui V. W. Tam C. C. (2011). Interim follow-up of a randomized controlled trial comparing Chinese style mind body (Tai Chi) and stretching exercises on cognitive function in subjects at risk of progressive cognitive decline. *Int. J. Geriatric Psychiatry* 26 733–740. 10.1002/gps.2602 21495078

[B13] Lam L. C. Chau R. C. Wong B. M. Fung A. W. Tam C. W. Leung G. T. (2012). A 1-year randomized controlled trial comparing mind body exercise (Tai Chi) with stretching and toning exercise on cognitive function in older Chinese adults at risk of cognitive decline. *J. Am. Med. Dir. Assoc.* 13 568.e51–568.e520. 10.1016/j.jamda.2012.03.008 22579072

[B14] Legault C. Jennings J. M. Katula J. A. Dagenbach D. Gaussoin S. A. Sink K. M. (2011). Designing clinical trials for assessing the effects of cognitive training and physical activity interventions on cognitive outcomes: the seniors health and activity research program pilot (SHARP-P) study, a randomized controlled trial. *BMC Geriatrics* 11:27. 10.1186/1471-2318-11-27 21615936 PMC3126708

[B15] Livingston G. Sommerlad A. Orgeta V. Costafreda S. G. Huntley J. Ames D. (2017). Dementia prevention, intervention, and care. *Lancet* 390 2673–2734. 28735855 10.1016/S0140-6736(17)31363-6

[B16] Ma D. Y. Wong C. H. Y. Leung G. T. Y. Fung A. W. T. Chan W. C. Lam L. C. W. (2017). Physical Exercise helped to maintain and restore functioning in chinese older adults with mild cognitive impairment: a 5-year prospective study of the hong kong memory and ageing prospective study (HK-MAPS). *J. Am. Med. Dir. Assoc.* 18 306–311. 10.1016/j.jamda.2016.10.003 27876478

[B17] Mann S. Beedie C. Jimenez A. (2014). Differential effects of aerobic exercise, resistance training and combined exercise modalities on cholesterol and the lipid profile: review, synthesis and recommendations. *Sports Med.* 44 211–221. 10.1007/s40279-013-0110-5 24174305 PMC3906547

[B18] Manton K. G. Gu X. Lamb V. L. (2006). Change in chronic disability from 1982 to 2004/2005 as measured by long-term changes in function and health in the U.S. elderly population. *Proc. Natl. Acad. Sci. U.S.A.* 103 18374–18379. 10.1073/pnas.0608483103 17101963 PMC1635981

[B19] Nasreddine Z. S. Phillips N. A. Bédirian V. Harbonneau S. Whitehead V. Collin I. (2005). The Montreal cognitive assessment, MoCA: a brief screening tool for mild cognitive impairment. *J. Am. Geriatrics Soc.* 53 695–699. 10.1111/j.1532-5415.2005.53221.x 15817019

[B20] O’Donovan G. Owen A. Bird S. R. Kearney E. M. Nevill A. M. Jones D. W. (2005). Changes in cardiorespiratory fitness and coronary heart disease risk factors following 24 wk of moderate- or high-intensity exercise of equal energy cost. *J. App. Physiol.* 98 1619–1625. 10.1152/japplphysiol.01310.2004 15640382

[B21] Pattyn N. Cornelissen V. A. Eshghi S. R. Vanhees L. (2013). The effect of exercise on the cardiovascular risk factors constituting the metabolic syndrome: a meta-analysis of controlled trials. *Sports Med.* 43 121–133. 10.1007/s40279-012-0003-z 23329606 PMC3693431

[B22] Prince M. Bryce R. Albanese E. Wimo A. Ribeiro W. Ferri C. P. (2013). The global prevalence of dementia: a systematic review and metaanalysis. *Alzheimers Dement.* 9 63.e2–75.e2. 10.1016/j.jalz.2012.11.007 23305823

[B23] Sink K. M. Espeland M. A. Castro C. M. Church T. Cohen R. Dodson J. A. (2015). Effect of a 24-month physical activity intervention vs health education on cognitive outcomes in sedentary older adults: the LIFE randomized trial. *Jama* 314 781–790. 10.1001/jama.2015.9617 26305648 PMC4698980

[B24] Sofi F. Valecchi D. Bacci D. Abbate R. Gensini G. F. Casini A. (2011). Physical activity and risk of cognitive decline: a meta-analysis of prospective studies. *J. Int. Med.* 269 107–117. 10.1111/j.1365-2796.2010.02281.x 20831630

[B25] Varma V. R. Chuang Y. F. Harris G. C. Tan E. J. Carlson M. C. (2015). Low-intensity daily walking activity is associated with hippocampal volume in older adults. *Hippocampus* 25 605–615. 10.1002/hipo.22397 25483019 PMC4425252

[B26] Xiao S. Li J. Tang M. Chen W. Bao F. Wang H. (2013). Methodology of Chin’s national study on the evaluation, early recognition, and treatment of psychological problems in the elderly: the China Longitudinal Aging Study (CLAS). *Shanghai Arch. Psychiatry* 25 91–98. 10.3969/j.issn.1002-0829.2013.02.005 24991140 PMC4054537

[B27] Yagci G. Yakut Y. Simsek E. (2018). The effects of exercise on perception of verticality in adolescent idiopathic scoliosis. *Physiother. Theory Pract.* 34 579–588. 10.1080/09593985.2017.1423429 29308950

[B28] Zheng G. Zhou W. Xia R. Tao J. Chen L. (2016). Aerobic exercises for cognition rehabilitation following stroke: a systematic review. *J. Stroke Cerebrovasc. Dis.* 25 2780–2789. 10.1016/j.jstrokecerebrovasdis.2016.07.035 27554073

